# On the Influence of the University Tenure on the Digital Pandemic Stress in Higher Education Faculty

**DOI:** 10.3390/bs13040335

**Published:** 2023-04-17

**Authors:** Álvaro Antón-Sancho, Diego Vergara, María Sánchez-Calvo, Pablo Fernández-Arias

**Affiliations:** Technology, Instruction and Design in Engineering and Education Research Group, Catholic University of Ávila, 05005 Ávila, Spain; alvaro.anton@ucavila.es (Á.A.-S.); maria.sanchez@ucavila.es (M.S.-C.); pablo.fernandezarias@ucavila.es (P.F.-A.)

**Keywords:** anxiety, digital competence, stress, digital technologies, survey

## Abstract

This paper conducts quantitative research on the levels of stress generated in Latin American professors due to the abrupt digitalization of higher education resulting from the COVID-19 pandemic. Specifically, the differences in this digital stress between professors from private and public universities are analyzed. For this purpose, a validated questionnaire has been used and passed to a sample of 750 professors from 20 different Latin American countries, whose responses have been statistically analyzed. As results, it has been obtained that there are no significant differences between the average levels of digital stress of professors of private and public universities due to the pandemic. However, the way in which this digital stress has affected Latin American professors according to gender and age is different depending on the university tenure. As a consequence, some implications and recommendations derived from the results are presented.

## 1. Introduction

### 1.1. Presentation and Approach

COVID-19, originating in the Chinese city of Wuhan, spread globally, being declared by the World Health Organization as a pandemic in March 2020, with catastrophic consequences on the world economy and people’s well-being [1,2]. This situation became a global challenge, with a significant burden on the health domain, as well as other sectors, leading to the adoption of strict containment measures to control this pandemic at national and international levels [3]. Specifically, in the educational field, relevant changes were adopted during this period, such as implementing emergency remote teaching, in which the content previously addressed in the classroom was taught outside the classroom, using the means and devices available at the time [4]. Attempts to give continuity to education through digital tools were not without difficulties, as professors had insufficient digital competence and inadequate technological equipment, which led to heterogeneous access and implementation [5], making visible the inequalities that lurk at the bottom of societies [6]. Faculty were forced to adapt to online teaching in an almost immediate way, highlighting the weaknesses of the educational system, based mainly on face-to-face interactions [7], and generating a disruption of classical pedagogy methods [8], with consequent stress and emotional exhaustion in the faculty [9]. The psychological effects of teaching during this period were determined by the relationship between work demands, available resources, teachers’ resilience levels and the fatigue generated by the situation [10], causing in them evident somatic repercussions, stress linked to change, and emotional exhaustion [9].

This situation of digital stress for faculty was experienced differently depending on certain aspects of the geographical location of professors, such as the level of digitization and technological development of the country of origin [11]. Specifically, in Latin America and the Caribbean, the region on which this paper focuses, the digitization process in higher education had to respond to the strong existing gaps in digital training and access to technologies of faculty for various reasons, including gender [12] or age [13,14]. The tenure of universities—public or private—is a particularly influential factor in the way this process of integration of digital technologies was carried out since the pandemic has generated inequalities due to connectivity difficulties and limited economic resources depending on each type of institution [15]. These inequalities are determined by the form of funding and the technical equipment available in each type of institution, suggesting that the affective and digital stress dimensions of faculty derived from the digitalization of higher education caused by the pandemic are strongly dependent on university tenure [11].

In this context, the exploration of the affective consequences of the digitalization process among Latin American university faculty should consider the differences in professors’ university tenure. To address this gap, this paper conducts research of the levels of stress regarding the use of digital technologies that the COVID-19 pandemic has caused among Latin American university professors. The main novelty of the work is the identification of the main differences in the impact of this digitization process on the development of digital stress among professors in private and public universities. Specifically, it analyzes the differences in the behavior of gender, age, and area of knowledge gaps in the levels of pandemic stress according to the university tenure of the professors.

### 1.2. Research Objectives

The general objective of this research is to quantitatively analyze the impact of the process of digitization of higher education teaching on the digital pandemic stress of professors in the Latin American and Caribbean region and to identify the differences in this regard between professors from private and public universities. In particular, the following specific research objectives will be achieved: (i) to describe the self-concept of digital competence of the participating professors, their assessment of the professional aspects linked to the process of digitization of teaching, and the digital pandemic stress they have suffered; (ii) to identify differences in the perceptions of the professors regarding the above dimensions according to the tenure of their respective universities; (iii) to study whether the behavior of the gender, age, and area of knowledge gaps in the above perceptions is different according to the university tenure.

## 2. Literature Review

### 2.1. Integration of Digital Technologies in Latin American Universities

The incorporation of digital technologies in the field of higher education is determined by the economic development of each country and conditions its level of digitization, determining the process of technological adaptation of the different countries to adjust to this new situation of virtual teaching [11,16]. The Global Innovation Index (GII) [17], developed by Cornell University and the Institut Européen d’Administration des Affaires (INSEAD, Fontainebleau, France), published by the World Intellectual Property Organization (WIPO, Geneva, Switzerland), highlights in its results that the Latin American region is an area in which the digitization process is, to a greater or lesser extent, uniform, which justifies the analysis of this region as the object of this study [7,11]. The process of the digital transformation of higher education in Latin America and the Caribbean is very slow compared to other regions [18]. In addition, faculty in Latin America and the Caribbean have had to face conditioning factors derived from the characteristics of the geography of their region, related to its great extension and the high dispersion of its population, in addition to unequal access to technological resources [19]. Difficulties in the inclusion of new technologies in university classrooms in Latin America and the Caribbean can be seen at different levels: the microsystem or teacher; the mesosystem or infrastructure conditions and formal and informal support of teachers; the exosystem or factors linked to the opinion, satisfaction, and experience of third parties; and the macrosystem or ministerial policies [20].

It is relevant to consider that higher education in Latin America has become universalized, expanding the number of students served, although with insufficient state funding [21]. Therefore, Latin America has been a pioneer region in the growth of private higher education, presenting countries with majority enrollments in institutions of this type, as is the case of Brazil and Chile [22], and gathering more than 50% of the total enrollment today in this private sector [23,24]. Focusing attention on the way in which university professors have faced this situation according to their university tenure, it is observed that professors from both public and private universities present a medium level of digital skills, high commitment, and medium levels of role stress, with the higher the level of stress, the lower the commitment, and the higher the commitment, the higher the level of digital skills [25]. On the other hand, university professors at private Latin American institutions with a medium or low level of innovation (depending on the GII of the corresponding countries) present a self-concept, in terms of work motivation, that is more optimistic than those at public universities, probably justified by the fact that private institutions invest more in digital learning resources and faculty training [26]. In the case of professors at public universities, the effects of the low budgets granted by the state are manifested in the precariousness in the implementation of infrastructure and equipment, as well as in the allocation of hourly pay for contracted professors, with the consequent discomfort that this causes [27]. In this sense, the literature confirms that Latin American private universities are the ones that have bet more decisively on the incorporation of digital technologies and, consequently, make a greater investment effort [28]. In both public and private institutions, low levels of academic performance have become an educational problem, although governments have certainly initiated changes that seek to reverse this reality within a context that contemplates a constructivist, investigative, and continuous evaluation system of the teaching–learning process in curricular planning [25]. In short, COVID-19 evidenced the need to provide relevant, interactive, and user-friendly digital teaching resources and content, constituting a turning point in the development of online content in the classroom [29]. Most studies related to digital transformation in higher education focus on the need to strengthen digital strategies to reinforce the use of technologies as part of the digital agenda in Latin America and the Caribbean [14]. In this task, university professors and the digital competencies that this task requires are directly involved.

### 2.2. Digital Competence of Faculty

The digital competence of professors can be defined as the ability to adapt to virtual learning environments applying physical and digital resources [30]. This digital competence of faculty does not only involve the use of classic digital tools such as the design of presentations through specific software or content sharing platforms. Far from that, the digital competence of university professors requires constant updating on their part in terms of the incorporation of new technological developments for teaching use, such as virtual reality tools, social networks, or artificial intelligence, among others [30,31]. The specificity of the digital competence of higher education professors lies in the fact that the use of these digital tools should favor, as far as possible, a self-regulated learning by students, which helps them to integrate, as realistically as possible, in a knowledge society that is strongly digitalized and highly globalized, professionalized, and competitive. This is what is known as Education 4.0 [30], which requires university professors to have the highest level of digital training possible, regardless of the area of knowledge in which they are experts.

All of the above requires the promotion of communication and dissemination of information in virtual environments, which translates into developing the ability to handle information, use it to build knowledge, learn to respond to problems and needs in daily practice, recognize the need for such information, analyze it, manage it, store it and transform it into knowledge, making it reach the students in a clear and concise manner [31]. This purpose requires collaboration and cooperation skills, promoting exchange to facilitate the construction of knowledge [32], constituting teamwork as a key tool for professors in their professional performance [33]. On the other hand, the basis of digital competence requires the interaction between content, pedagogy, and technological knowledge [34]. Thus, a vital factor in the digital transformation of educational institutions, and therefore essential as a complement to their digital competencies, is the vision of their faculty as networked leaders, understanding that digital transformation can be determined by the clarity of the strategy employed by professors who support a culture capable of fostering and changing ideas and practices [35]. This web leadership involves overcoming difficulties and obstacles arising from inadequacies related to their own technological knowledge and skills of faculty to guide digital development within their classrooms, provide appropriate guidance, and facilitate the adjusted use of technology within the learning environment [36]. This requires emotional work in the use of digital tools to compensate for the anxieties that the experience of incorporating new technologies into teaching practice may raise [37].

One of the ways to test the degree of digitalization of teaching processes in higher education is to assess the self-concept of the digital skills of the teaching staff [4,26,29]. This allows, on the one hand, to analyze the degree of adherence of professors to the use of digital technologies required by Education 4.0 and, on the other hand, to detect the difficulties or shortcomings that professors encounter in this process.

Understood in this way, the literature shows that, in general, university faculty have low levels of digital skills, which are insufficient to meet the challenges required by the process of integrating digital technologies in higher education. This has been verified in European [5,38], American [26,39], and Asian [40,41] faculty. Latin American university professors are aware of this competency gap [26] and attribute it, in general, to two main reasons: (i) a lack of training in digitization and the development of adequate techno-pedagogical competencies [26]; and (ii) a lack of investment by universities and states in technical resources, human capital, and adequate spaces for a correct integration of these digital technologies [42].

### 2.3. Digital Stress among Faculty Members

The difficult educational situation generated and the impact it had on university faculty and students were evident [8,43] and affected the occupational health and stress of faculty, due to difficulties in the pedagogical use of technologies [44]. This situation significantly worsened a situation of emotional stress classically linked to university teaching which existed before the pandemic due to circumstances such as pressure due to high workloads, multiplicity of tasks, time constraints [45], unbalanced integration between work and personal life, limited autonomy, excessive administrative duties, tensions with peers and managers, role conflicts and ambiguity, management of innovation and change, the burden of emotional labor, fear of losing control of the classroom, fear of evaluation, and low professional self-esteem, among others [46]. All of this leads to the existence of disorders among teachers, such as chronic stress, emotional burnout, or burnout syndrome [47]. The latter is a disorder made up of a plurality of negative psychological experiences and maladaptive behaviors caused by prolonged tensions which manifest themselves through unfavorable feelings and emotional attitudes towards work and students [48]. In the case of higher education professors, the development of feelings of stress and anxiety due to digitalization is linked to the so-called digital pressure, which consists of the responsibility of leading this digitalizing process, designing didactic strategies that incorporate the new digital technologies, and the need to be in constant contact with the new digital technologies and satisfy the requirements of Education 4.0 [49].

The triggers for the high levels of stress caused in professors by the abrupt digitalization process derived from the pandemic have been very diverse [50,51]. Some of them are derived from the pressure imposed by the use of online educational methods, such as increased working hours, difficulties due to the lack of physical contact, problems in reconciling personal and work life [52,53], the complexity of digital migration due to technical circumstances [26], the inadequate equipment to cope with online teaching, the limited digital competence of teachers and students [5], the absence of competencies related to the evaluation of educational practice [54], the weakness of the online teaching infrastructure, the environment not conducive to home learning for some students, and how all of these affected academic equity and excellence [42].

In short, university professors were forced to recognize a low or medium-low digital competence conditioned by multiple aspects, among which were the inability to provide solutions to problems using technologies, to show ability to work with a network of contacts, and to make use of 2.0 tools to evaluate their students and the lack of appropriate strategies to evaluate their educational practice [55]. On the other hand, age was also considered a determining factor, as the professors’ experience constituted a factor that positively impacted academic organizational effectiveness due to greater job satisfaction generated by higher financial well-being, having full-time and permanent work, and occupational prestige [13,55]. Furthermore, among faculty, as among all professionals, the prospect of retirement acts as a cause of decreased levels of job anxiety [56,57]. Thus, younger professors manifested greater deterioration in mental health than their older colleagues [58]. In this sense, the impact of the pandemic on younger professors was combined with the inexperience of their age [59], which explains why an upward spike in the levels of anxiety and depression was observed among them.

Indeed, COVID-19 aggravated the effect of a family of symptoms in the somatic area of university teachers, stress generated by change, and emotional exhaustion [9]. The stress generated in Latin American teachers by this situation has been increased by the generalized assessment that the training received from their institutions to integrate new technologies into their work environment has been insufficient [60].

The specialized literature indicates that continuous training in digital competence is essential to sustain updated digital skills on the part of teachers, improve their educational effectiveness, and to reduce the digital gaps between them [61,62]. This training has the effect of increasing the security in the use of digital technologies by teachers, encourages their didactic use in their lectures, reduces digital pressure, and reduces certain digital gaps, such as gender gaps [63]. Therefore, it is advisable to intensify the efforts that both public and private universities make to improve the digital competence of teachers [26], to promote their pedagogical adaptation to various tools, platforms and applications, adapting to the particularities of each area of knowledge taught [64], as well as to develop interventions that promote the mental comfort of professors, facilitating emotional regulation and the development of digital skills [65]. This implies that educational administrations, governments, and authorities should seek funding options, both internal and external, to keep their technological infrastructures as up to date as possible for the benefit of students and professors [66].

## 3. Materials and Methods

### 3.1. Participants

The sampling process was non-probabilistic by convenience. The target population consisted of university professors from the Latin American and Caribbean region who attended a training session on digital competence in teaching and the use of ICT in higher education given by the authors and repeated every two weeks between January and June 2022 (a total of 785 professors). The term professor used here covers a broad range of higher education teachers, including teaching assistants, assistant professors, associate professors, and full professors. The objectives of this training session were: (i) to present the conceptual framework of digital competence in teaching; (ii) to describe the different areas of teaching activity and the different digital tools to address them; and (iii) to develop practical cases of ICT application in higher education. After the training session, the authors sent the questionnaire that was used as a research instrument to the attendees by e-mail. The criteria for inclusion in the study were: (i) to be an active professor at a university in the Latin American and Caribbean region with at least three years’ seniority (to ensure that the participant had teaching experience prior to the pandemic); and (ii) to have attended the training session given by the authors. The response collection process followed the stipulations of the Declaration of Helsinki. No data were collected that would allow identification of the participants, and their participation was voluntary, free, anonymous, and based on informed and express consent. Finally, a total of 750 professors participated in the study.

### 3.2. Variables and Instrument

In this research, university tenure is defined as the main explanatory variable—nominal dichotomous, whose possible values are public or private—and as secondary explanatory variables: (i) gender—nominal dichotomous with values female or male, (ii) age—continuous quantitative variable, and (iii) area of knowledge—nominal polytomous, whose possible values are Arts and Humanities, Sciences, Health Sciences, Social and Legal Sciences, or Engineering and Architecture. The areas of knowledge are delimited according to UNESCO’s integrated classification of areas of knowledge [67], integrating the area of education within the area of social and legal sciences. The explanatory variables are: (i) the self-concept of digital competence; (ii) the assessment of the professional aspects linked to the process of digitalization of higher education; and (iii) the level of digital pandemic stress expressed by the participants. The three variables explained have been defined in terms of ratings and have been measured in 1–5 Likert scales, where 1 means no rating, 2 is a low rating, 3 is an intermediate rating, 4 is a high rating, 5 is a very high rating.

A validated questionnaire was used as a research instrument to measure the self-concept of digital competence of university professors, the assessment of the importance of professional aspects linked to teaching in the process of integrating ICT in lectures, and digital pandemic stress [11]. The questionnaire consists of 22 questions that, according to the results of the factor analysis, are grouped into three families: (i) self-concept of digital concept—items 1–11, on digital skills, adaptation to digital learning environments, capacity for continuous learning, communication skills, creativity when using digital resources, knowledge of information management, network leadership, orientation to students, resilience, teamwork, and strategic vision; (ii) assessment of professional aspects related to the digitalization process in higher education—items 12–14, on the support of the university, the technical equipment, and the training received in digital matters; and (iii) assessment of digital pandemic stress—items 15–22, on insecurity, anxiety, feeling that difficulties are increasing, feeling of inability to achieve the objectives of digitization, irritability, nervousness, fear of contagion, and feeling of not being in control of the situation. Thus designed, it has been shown that the instrument, which is structured in the three scales or families of questions which measure the three defined dependent variables, is reliable [11].

### 3.3. Design and Statistical Analyses

The present work consists of descriptive quantitative research on the self-concept of digital competence, the assessment of professional aspects related to the digitization of higher education, and the levels of digital pandemic stress of a sample of university professors in the Latin American and Caribbean region. The research was conducted in four phases: (i) determination of objectives, definition of variables, and choice of the research instrument; (ii) delivery of the initial training session and sampling; (iii) data collection; (iv) statistical analysis of responses and drawing conclusions.

For the statistical analysis, descriptive statistics were obtained for the responses to the different families of questions, both in general and by differentiating by university tenure. The *t*-test was used to identify significant differences in the mean responses between the professors of the two types of universities. Likewise, the multifactor analysis of variance test (MANOVA) was used to study whether there are significant differences between the distributions of responses by gender and areas of knowledge between the two possible university tenures. Finally, linear and polynomial regression models of degrees greater than 1 have been constructed between the different variables explained and the age variable, to describe how age can explain each of the explained variables analyzed.

## 4. Results

### 4.1. Description of the Sample of Participants

**Figure 1:**
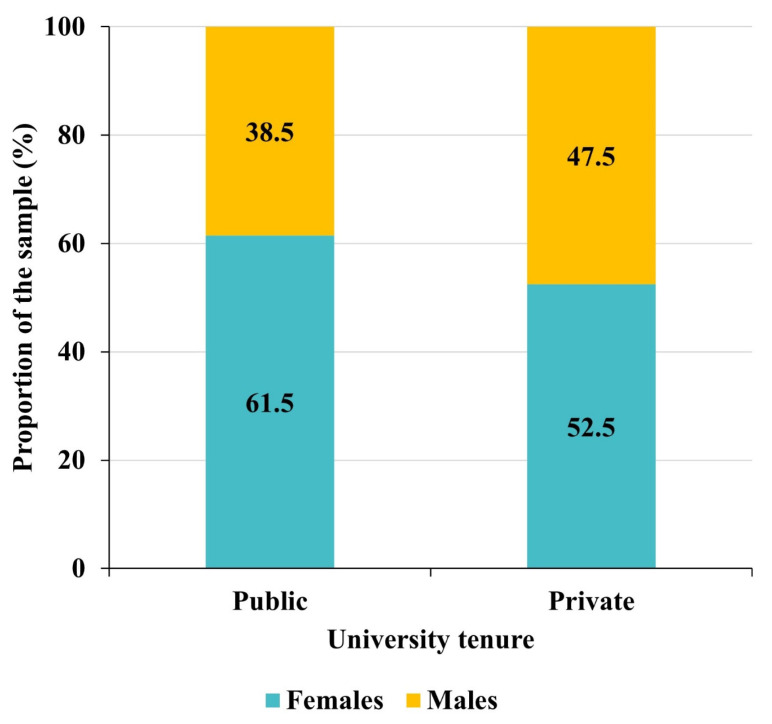
Distribution of participants by gender and university tenure.

A total of 750 Latin American professors (42.4% male, 57.6% female; mean age 47.15 years, sd = 11.23 and median 50 years) participated in this research. As shown in Table 1, the distribution of participants by country is not homogeneous (χ^2^ = 1026.5, *p*-value < 0.0001). There is a slight majority of professors from public universities (56.8%) versus private (43.2%), which generates a certain bias that turns out to be statistically significant (χ^2^ = 13.8720, *p*-value = 0.0002). The gender distribution is different in the two types of universities since the superiority of the proportion of females is greater in public universities than in private universities (Figure 1), with this difference in the gender distributions between the two types of centers being statistically significant (χ^2^ = 6.1488, *p*-value = 0.0132).

**Figure 2:**
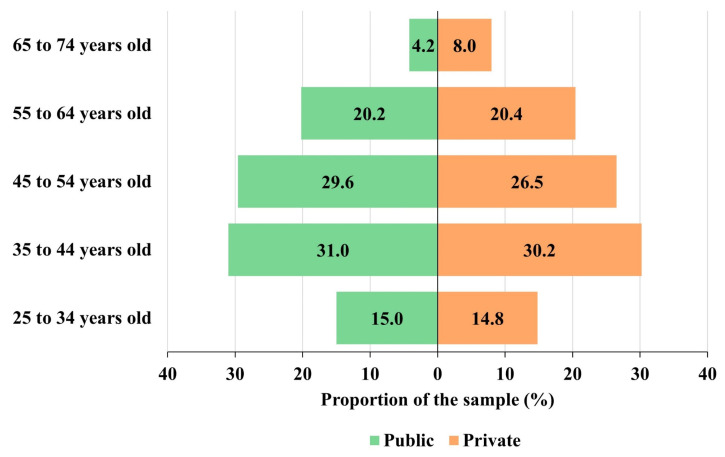
Distribution of participants by age and university tenure.

The mean age of participating professors from public universities is 46.76 years old (sd = 10.86), while professors from private universities have a mean age of 47.65 years (sd = 11.70). Therefore, the ages of the participating professors are slightly more dispersed in private universities, which is probably because they have a smaller proportion of professors between 35 and 54 years of age than public universities, while the proportion of older professors (over 65 years old) is greater than in public universities (Figure 2). This fact probably also explains why the mean age in private universities is almost one year higher than that of professors in public universities.

**Figure 3:**
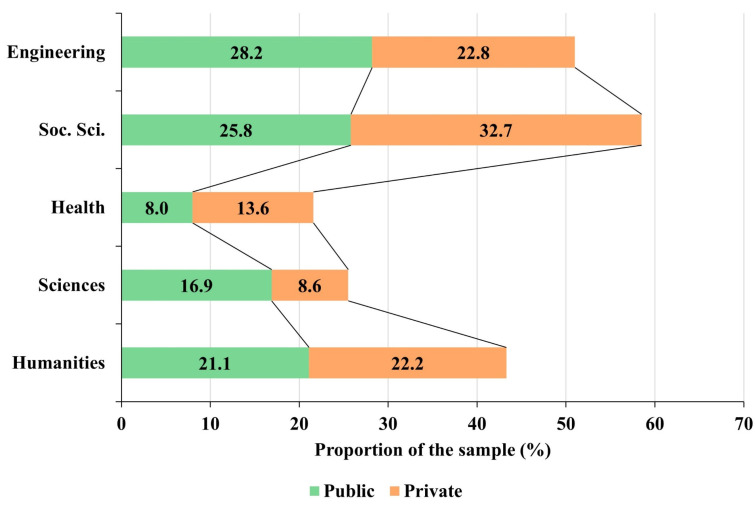
Distribution of participants by area of knowledge and university tenure.

The majority areas of knowledge are Humanities, Social Sciences, and Engineering and Architecture in both types of centers. However, there is a higher percentage representation of professors of Sciences and Engineering in public universities than in private universities, while in the latter, there is a notably higher percentage representation of professors of Health Sciences and Social Sciences than in public universities (Figure 3). The representation of the Humanities area differs by only one percentage point between the two types of centers. The distribution of participants by subject area therefore differs significantly between public and private universities (χ^2^ = 20.1240, *p*-value = 0.0005).

### 4.2. Analysis of Responses

The responses to the questions that fall into each of the three families described above—self-concept of digital competence, assessment of professional aspects liked to digitalization, and assessment of digital pandemic stress—were grouped and the means and variances of these families of responses were computed (Table 2). The participants express an intermediate average self-concept of digital competence (between 3 and 4 out of 5). The valuation of their pandemic digital stress is intermediate (under 3 out of 5) but has the greatest dispersion, which implies that this is the variable with the greatest unevenness among the participants. The average rating of the professional aspects of the digitalization of teaching is also intermediate (between 3 and 4 out of 5).

From the computation of the Pearson correlation coefficients, it can be deduced that there is a moderate positive correlation between the valuation of the professional aspects and the self-concept of digital competence, but that the levels of pandemic digital stress are not linearly related to the rest of the variables analyzed (Table 3). In particular, pandemic digital stress does not have a significant linear relationship with the self-concept of digital competence among the participating teachers.

The bilateral *t*-test for independent samples carried out with Welch’s correction, without assuming equality of variances, shows that the participating teachers from private universities show a higher self-concept of their digital competence and give better average ratings of the professional aspects linked to digitalization than their colleagues from public universities (Table 4). However, there are no significant differences between the levels of digital pandemic stress of the two groups (Table 4).

The data reveal that there are no significant gender gaps in digital competence self-concept (χ^2^ = 1.2259, *p*-value = 0.2682) in either private or public universities, but there are significant gender gaps in digital pandemic stress (χ^2^ = 5.1793, *p*-value = 0.0229), which is higher among female professors in both types of universities, although the widest gap is in public universities (Table 5). The MANOVA test statistics show that it can be assumed that the behavior of gender differences in public and private universities with respect to the self-concept of digital competence and digital pandemic stress is analogous. However, regarding the assessment of professional aspects, the behavior of gender differences is different in the two types of universities (Table 5). Indeed, in public universities, males give higher mean ratings than females (although the difference is small, only 1.24%), while in private universities, females give higher mean ratings than males, the difference being wider in this case (of 4.75%) (Table 5).

To facilitate the analysis of the dependence of the responses on the age variable, linear regression models of the two families of responses with respect to the age of the participants have been elaborated. These models are statistically significant for the responses on digital competence (t = −9.79, *p*-value < 0.0001) and digital pandemic stress (t = −5.05, *p*-value < 0.0001), but the R^2^ values, which indicate the variability explained by the model, are very small (R^2^ = 0.0115 for digital competence and R^2^ = 0.0042 for digital stress). This means that the model is not useful for reporting exact values, but it does significantly explain the trends of growth or decrease of responses with age. Thus, it can be assumed that the self-concept ratings of digital competence and digital stress decrease as age increases since the slopes of the corresponding linear models are negative and statistically significant (−0.0089 for digital competence and −0.0072 for digital pandemic stress).

**Figure 4:**
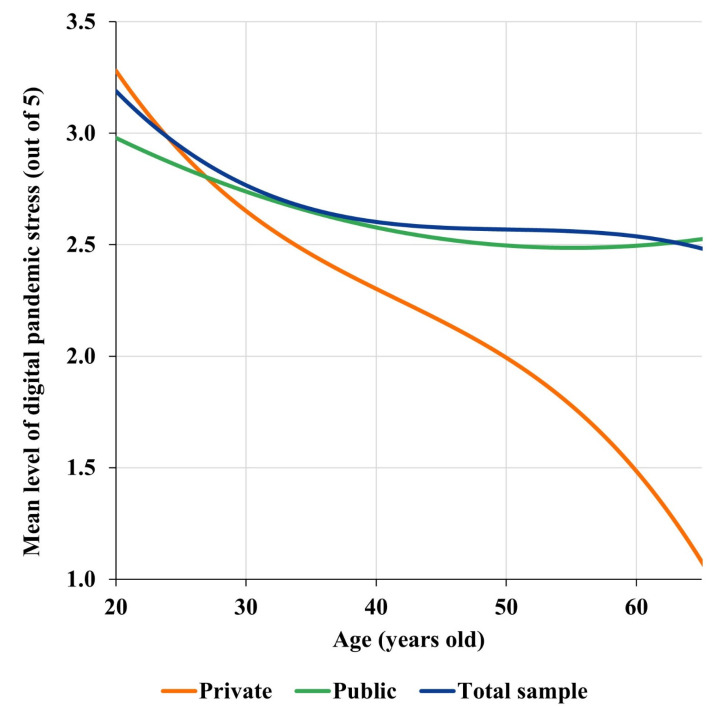
Representation of the regression polynomials of the digital pandemic stress ratings with respect to the age of the participants in private and public universities and in the total sample.

In the case of the digital pandemic stress response family, a better mathematical fit is obtained by using polynomial regression models using higher degree polynomials (specifically, degree 3). The variability explained is still very small (R^2^ = 0.0051), so that the model does not accurately fit the responses, but the model turns out to be significant (t = −2.15, *p*-value = 0.0315), thus explaining the trends in the behavior of the responses and, in fact, refining the information provided by the linear models. Indeed, this model confirms the decreasing trend of stress with increasing age but adds the fact that this decrease is much more accelerated among the youngest participants (approximately under 40 years old) and among the longest-lived (approximately over 55) than among middle-aged participants (approximately between 40 and 55 years), an age range in which the expressed stress levels behave in an approximately stationary way (Figure 4). This same mathematical adjustment also allows to significantly explain the trends of digital pandemic stress as a function of age in private universities (t = −2.38, *p*-value = 0.0172, with degree 3 polynomial) and in public universities (t = 2.60, *p*-value = 0.0094, with quadratic polynomial), although the variability of the responses again is weakly explained (R^2^ = 0.0125 for private universities and R^2^ = 0.0032 for public universities). Thus, in private universities digital pandemic stress decreases with increasing age, and the decrease is more accelerated in those younger than approximately 40 years old and in those older than around 55 years old. That is, a phenomenon like that described in general, without distinguishing between private and public universities, occurs. However, the decrease in stress among public university professors is lower and less accelerated as age increases until around 55 years of age—which corresponds to the vertex of the parabola—when stress begins to increase (Figure 4).

Participants from private universities express a higher average self-concept of digital competence than those from public universities in all areas of knowledge, although this superiority is wider or less wide depending on the area of knowledge (Table 6). In private universities, it is the Engineering professors who express a higher self-concept of digital competence, while in public universities, it is the Humanities professors who are more optimistic about their digital competence (F-statistic = 7.69, *p*-value < 0.0001). Regarding the professional aspects linked to digitization, professors at private universities value them more highly than their colleagues at public universities in all areas of knowledge, especially in Humanities, Sciences, and Social Sciences (F-statistic = 3.88, *p*-value = 0.0038). Thus, contrary to what happens with the self-concept of digital competence, in private universities, the Humanities professors are the ones who best value the professional aspects, while in public universities, it is the Engineering professors who give the highest ratings (Table 6). Finally, the digital pandemic stress of professors from private universities is higher than that of professors from public universities in Humanities, while in Social Sciences and Health Sciences, professors from public universities express greater digital pandemic stress than their colleagues from private universities (F-statistic = 7.45, *p*-value < 0.0001). In the areas of Science and Engineering, there are no significant differences by university tenure in terms of digital pandemic stress (Table 6).

## 5. Discussion

Participating Latin American and Caribbean professors express intermediate–high self-concepts of digital competence (almost 4 out of 5), with an intermediate level of digital stress (slightly above 2.5 out of 5). These results are consistent with the limited levels of digital skills that the literature attributes to professors both in general [5] and linked to some specific aspects of the teaching activity, mainly student assessment, which is the activity in which professors manifest a lower ability [54]. Usually, the literature correlates low levels of faculty digital skills with the scarcity of economic investment in adequate infrastructure by universities [11,42]. The results obtained here contradict this observation since the ratings on self-concept of digital competence and professional aspects linked to digitization turn out to be statistically independent (Table 3 and Table 4). Therefore, the results lead us to think that teachers link their digital competence more to their digital training, as other studies suggest [26], although this conclusion should be contrasted with subsequent specific studies.

A moderate but positive correlation was also found to exist between self-concept of digital competence and expressed levels of digital stress (Table 3). This result is in apparent contradiction with the effect of reducing digital stress that the literature attributes to professors’ digital training [61]. However, the results obtained can be explained if it is understood that a higher self-concept of digital competence probably leads to a greater awareness of the digital pressure felt by professors which can lead to greater perceived digital stress [49]. However, a more in-depth correlational study would be needed to confirm this hypothesis.

The results obtained also allow to assume that professors in Latin American private universities have, in general, better self-concepts of their digital competence than professors in public universities, and they also value better than the latter the professional aspects linked to the digitalization of higher education (Table 4). Private universities in the Latin American region probably have a higher proportion of distance students than public universities, which may explain why they make a greater digitization effort, both in terms of equipment and faculty training [14,15]. This would justify the fact that both the results obtained here and those of other studies systematically show that professors at private universities claim to have better digital skills than those at public universities [26]. This superiority can be explained by the higher level of integration of digital technologies that private universities in Latin America are experiencing [25,28], the precariousness and obsolescence of the infrastructure and equipment of public institutions in the region [27], and by the greater investment that private universities make in digital training of faculty [26].

Regarding the levels of digital stress, the results obtained are in line with the levels reported by other studies carried out in different geographical regions throughout the world [8,44], so it does not seem that the geographical variable is particularly explanatory in this regard. However, it has been observed that the variability of Latin American and Caribbean professors’ responses in terms of pandemic digital stress levels is high compared to the other response families (1.25 out of 5), indicating that there is much divergence among professors’ stress levels. This strong variability is consistent with the results of other work on pandemic digital stress carried out on samples of professors from more restricted geographical areas such as Venezuela [51]. The originality and novelty of the present work lies in the finding that strong unevenness of pandemic digital stress is found throughout the Latin American and Caribbean region.

In contrast to the ratings of the professional aspects, a moderate but significant positive correlation was found between the participants’ levels of digital stress and their self-concept of digital competence (Table 3 and Table 4). In other words, a higher self-concept of digital competence was found to be linked to more intense digital stress, which is a novel and original contribution of the present study. The literature explains that the impact of the pandemic at the level of digitization of higher education was to make teachers responsible for providing the technical means so that the academic pace would not suffer [11,53]. Therefore, a higher self-concept of digital competence, which entails a greater awareness of this responsibility, may explain the higher level of digital stress observed.

Despite the better self-concepts on digital competence and the better assessments of the professional dimensions of the digitization process observed among professors at private universities, no significant differences in the level of pandemic digital stress were identified between professors at private and public universities (Table 4). It is also found that female professors both in private and public universities manifest slightly higher levels of pandemic digital stress than male professors, with no significant difference in the behavior of this gender gap by university tenure (Table 5). This is consistent with the results obtained by previous work in the specific setting of Venezuela, but not with the results obtained in countries with low levels of digitization in Latin America, such as Guatemala, Honduras, and Bolivia [51]. Therefore, the results suggest that there is a strong variability between countries with respect to the behavior of gender gaps in private and public universities, which offers the results presented here as an average. It is therefore advisable to carry out a subsequent comparative study among the countries of Latin America and the Caribbean to further explore the results obtained here.

**Figure 5:**
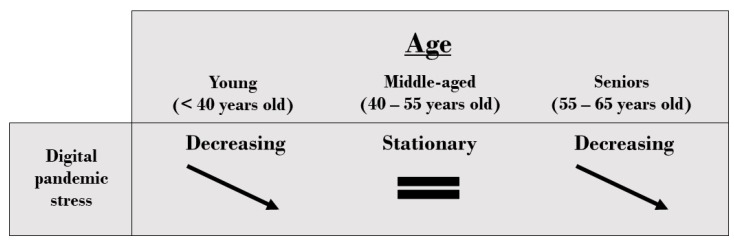
Overview of the behavior of age gaps in the levels of pandemic digital stress among Latin American professors.

In general, a decrease in digital pandemic stress is observed as the age of Latin American professors increases. The decrease is especially accelerated among those under 40 years of age and those over 55 years of age, and between 40 and 55 years of age it remains approximately stationary (Figure 5). In contrast to the gender variable, the behavior of the level of digital pandemic stress with respect to the age of the participants is significantly different according to university tenure. In particular, the intensity of pandemic stress decreases with increasing age among private university professors, while among public university professors, this stress increases among senior professors (Figure 4).

This result is original and novel in the literature and proves that the process of integration of digital technologies in universities in Latin America and the Caribbean is being carried out in a way that corrects the digital divide that exists in the region and that shows that the digitization of older professors is, in general, weaker [7]. In addition, the results obtained here with respect to private university professors are consistent with works that predict that the higher level of general self-confidence that older professors have [58] and their greater job stability [13,55] act as a containment mechanism for the level of digital stress. Likewise, although there is no specific analysis in the literature of professors’ digital pandemic stress behavior as a function of age, there are studies that show that, in general, middle-aged workers have high levels of stress due to work pressure, and that these stress levels decrease from 55 years [57] or 60 years of age [56], which is mainly motivated by the prospect of an approaching retirement [56]. There are also studies that show that there is a high peak of work stress among young people starting their professional life due to their lack of experience, which causes them to develop a strong insecurity when facing their work [59]. Precisely, in this study, it has been found that these phenomena reported in the previous literature—high levels of work stress among the youngest and a decrease among the oldest—occur in an analogous way with respect to digital pandemic stress among Latin American professors, for whom, moreover, an intermediate age range can be distinguished—approximately between 40 and 55 years of age—in which the levels of pandemic stress are stationary. Consequently, the results show that the behavior of digital pandemic stress with respect to the age of Latin American professors can be explained by considering three age brackets—young professors, under 40 years; middle-aged professors, from 40 to 55 years; and senior professors, from 55 to 65 years—so that the strongest decreases in stress occur in the first and last brackets. Furthermore, it has been found that this dependence on the age of the professors is different for private and public university professors, the latter seeming to have made less effort to digitally integrate the elderly, which translates into a rise in their levels of digital stress.

Finally, it has been shown that university tenure is also an explanatory variable of the way in which digital pandemic stress has affected the different areas of knowledge. In particular, the professors at public universities who express the greatest digital pandemic stress are those in Health and Social Sciences and Engineering, while in public universities, the greatest digital pandemic stress is found among professors in Humanities. Previous literature had already found that digital stress is more prevalent among professors in areas that, in principle, make less frequent use of digital technologies, such as the Humanities [7]. The novelty of the results obtained here lies in identifying that university tenure has a decisive influence on the way in which this digital stress affects the different areas of knowledge. In fact, in private universities, which are, in general, more intensely digitized than public universities [28], the general trend shown by previous literature is broken and a higher level of pandemic digital stress of professors in areas, a priori, highly digitized, such as Health Sciences or Engineering, is found.

## 6. Limitations and Lines of Future Research

In the distribution of the sample of participants there is a slight bias by university tenure and gender. Consequently, it is proposed to carry out a study like the one carried out here, in which the sample size is increased to ensure that it is distributed homogeneously by gender and university tenure. In this way, it will be possible to contrast the results obtained. It is also proposed to complete the study with a qualitative study, because this would make it possible to identify the underlying reasons for the gaps identified and the correlations observed. In this work, it has not been possible to carry out a comparative analysis by country due to the strong bias in the distribution of the participants in this respect. It is proposed to carry out a comparative study between countries, to test the influence of geographic location on the variables studied, mainly on the levels of digital pandemic stress and the variability of these levels, and the explanatory nature of factors such as the level of digitization of the specific country measured through the GII. It is also proposed to carry out a correlational study to explore the relationship of dependence that exists between the levels of general stress and digital stress, as well as the identification of other academic variables that may be explaining the levels of digital pandemic stress, such as the area of knowledge of the professors. Likewise, it would be important to study the impact of digital competence training plans provided by universities on the digital stress levels of professors.

## 7. Conclusions

Professors from Latin American and Caribbean universities express having an intermediate–high self-concept of digital skills (above 3.5 out of 5) and intermediate levels of digital pandemic stress (above 2.5 out of 5). There is a moderate, but significant, positive influence of digital skills on the level of digital pandemic stress, so that professors with a higher self-concept of digital skills suffer more intense digital stress. No significant differences are identified between the mean levels of digital pandemic stress of professors at private and public universities, although professors at private universities have a self-concept of digital competence 6.1% higher than that of professors at public universities and a valuation of professional aspects linked to digitalization almost 13.3% higher than that of their colleagues at public universities. Moreover, in both types of universities, female professors report slightly higher levels of digital pandemic stress than male professors, although the difference with males is greater in public universities (around 5% in public universities and 0.7% in private universities).

The age of Latin American professors is an influential variable in the behavior of their digital pandemic stress levels. Specifically, digital stress decreases, in general, with increasing age. However, among professors at private universities, this decrease is more accelerated among professors younger than 40 years and older than 55 years, while it is approximately stationary among professors aged 40–55 years. In contrast, the digital stress of public university professors decreases with age until the age of 55, at which point digital stress begins an upward trend.

The main implications of this study are as follows: (i) it is necessary to reinforce the funding allocated by universities to teacher training in digital competence and the development of techno-pedagogical skills; (ii) in the process of integrating digital technologies, it is necessary to carry out measures to especially favor the incorporation of female professors, especially in public universities, where females present higher levels of digital stress; and (iii) to carry out specific training for older professors in order to incorporate them adequately into the digital integration process, especially in public universities, where the digital divide is notably more pronounced with respect to the levels of digital pandemic stress.

## Figures and Tables

**Table 1:** Distribution of participants by country.

Country	Number of Participants	Proportion of Participants (%)
Argentina	149	19.87
Mexico	122	16.27
Ecuador	101	13.47
Venezuela	80	10.67
Peru	79	10.53
Colombia	66	8.80
Chile	23	3.07
Nicaragua	18	2.40
Brazil	16	2.13
Uruguay	14	1.87
Honduras	11	1.46
El Salvador	10	1.33
Panama	10	1.33
Dominican Republic	9	1.20
Puerto Rico	9	1.20
Bolivia	8	1.07
Cuba	8	1.07
Paraguay	8	1.07
Guatemala	5	0.66
Costa Rica	4	0.53

**Table 2:** Descriptive statistics of the responses of the different families of questions.

	Mean (Out of 5)	Standard Deviation (Out of 5)
Digital competence	3.86	0.94
Professional aspects	3.43	1.17
Digital stress	2.59	1.25

**Table 3:** Pearson correlations of the responses of the different families of questions.

	Digital Competence	Professional Aspects	Digital Stress
Digital competence	1	0.0008	0.4398 *
Professional aspects		1	0.0249
Digital stress			1

* *p* < 0.05.

**Table 4:** Mean responses (out of 5) by university tenure and statistics of the *t*-test of comparison of means for independent samples with the Welch’s correction.

	Public	Private	t-Statistic	*p*-Value
Digital competence	3.76	3.99	11.2980	<0.0001 *
Professional aspects	3.24	3.67	8.7833	<0.0001 *
Digital stress	2.61	2.57	−1.2465	0.2126

* *p* < 0.05.

**Table 5:** Mean responses (out of 5) distinguished by university tenure and gender, and MANOVA test statistics.

	Public		Private		t-Statistic	*p*-Value
	Females	Males	Females	Males		
Digital competence	3.76	3.76	4.02	3.96	1.9966	0.1577
Professional aspects	3.23	3.27	3.75	3.58	4.4015	0.0360 *
Digital stress	2.66	2.53	2.58	2.56	2.6501	0.1036

* *p* < 0.05.

**Table 6:** Mean responses (out of 5) of the families of questions distinguished by university tenure and gender, and MANOVA test statistics.

	Digital Competence		Professional Aspects		Digital Stress	
	Public	Private	Public	Private	Public	Private
Humanities	3.96	4.02	3.22	3.88	2.50	2.73
Sciences	3.63	4.05	3.22	3.71	2.50	2.54
Health	3.41	3.64	3.14	3.33	2.61	2.36
Social Sciences	3.73	4.06	3.19	3.74	2.70	2.48
Engineering	3.82	4.07	3.36	3.54	2.66	2.67

## Data Availability

The data are not public. They are available upon a reasonable request to the corresponding author.
